# A Novel Bearing Multi-Fault Diagnosis Approach Based on Weighted Permutation Entropy and an Improved SVM Ensemble Classifier

**DOI:** 10.3390/s18061934

**Published:** 2018-06-14

**Authors:** Shenghan Zhou, Silin Qian, Wenbing Chang, Yiyong Xiao, Yang Cheng

**Affiliations:** 1 School of Reliability and Systems Engineering, Beihang University, Beijing 100191, China; zhoush@buaa.edu.cn (S.Z.); qiansilin@buaa.edu.cn (S.Q.); xiaoyiyong@buaa.edu.cn (Y.X.); 2 Center for Industrial Production, Aalborg University, 9220 Aalborg, Denmark; cy@business.aau.dk

**Keywords:** rolling bearing, fault diagnosis, WPE, SVM ensemble classifier, hybrid voting strategy

## Abstract

Timely and accurate state detection and fault diagnosis of rolling element bearings are very critical to ensuring the reliability of rotating machinery. This paper proposes a novel method of rolling bearing fault diagnosis based on a combination of ensemble empirical mode decomposition (EEMD), weighted permutation entropy (WPE) and an improved support vector machine (SVM) ensemble classifier. A hybrid voting (HV) strategy that combines SVM-based classifiers and cloud similarity measurement (CSM) was employed to improve the classification accuracy. First, the WPE value of the bearing vibration signal was calculated to detect the fault. Secondly, if a bearing fault occurred, the vibration signal was decomposed into a set of intrinsic mode functions (IMFs) by EEMD. The WPE values of the first several IMFs were calculated to form the fault feature vectors. Then, the SVM ensemble classifier was composed of binary SVM and the HV strategy to identify the bearing multi-fault types. Finally, the proposed model was fully evaluated by experiments and comparative studies. The results demonstrate that the proposed method can effectively detect bearing faults and maintain a high accuracy rate of fault recognition when a small number of training samples are available.

## 1. Introduction

Faults in rotating machinery mainly include bearing defects, stator faults, rotor faults or eccentricity. According to statistics, nearly 50% of the faults of rotating machinery are related to bearings [1]. In order to ensure the high reliability of bearings and reduce the downtime of rotating machinery, it is extremely important to detect and identify bearing faults quickly and accurately [2]. The rolling bearing fault diagnosis method has always been a research hotspot. The vibration signals of rolling bearings often contain important information about the running state. When the bearing fails, the impact caused by the fault will occur in the vibration signal [3,4]. Therefore, the most common application of the bearing fault diagnosis method is to use the pattern recognition method to identify the fault by extracting the fault features of the bearing vibration signal [5]. However, the rolling bearing vibration signal is nonlinear and non-stationary. It is easily affected by the background noise and other moving parts during the transmission process, which makes it difficult to extract the fault features from the original vibration signal, and the accuracy of the fault diagnosis is seriously affected. Traditional time-frequency analysis methods have been used in bearing fault diagnosis and have achieved corresponding results, such as short-time Fourier transform and wavelet transform. However, all these methods have defects in the lack of adaptive ability for bearing vibration signal decomposition [6,7]. For complex fault vibration signals, relying only on subjectively setting parameters to decompose signals may cause the omission of fault feature information and seriously affect the performance of fault diagnosis [2].

Empirical mode decomposition (EMD) is one of the most representative adaptive time-frequency analysis methods, and has been widely used in sensor signal processing, mechanical fault diagnosis and other fields. However, the existence of mode mixing in EMD will affect the effect of signal decomposition, resulting in the instability of the decomposition results [8]. In order to improve the drawback of the mode mixing, Wu and Huang [9] proposed an effective noise-assisted data analysis method named ensemble empirical mode decomposition (EEMD), which added Gaussian white noise to the signal for multiple EMD decomposition. For bearing vibration signals containing rich information and complex components, the EEMD adaptively decomposes bearing vibration signals into a series of intrinsic mode functions (IMFs), which reduces the interference and coupling between the different fault signal feature information and helps highlight the deeper information of the bearing operating status.

For the nonlinear dynamic characteristics of the bearing vibration signal, various complexity measurement methods are utilized to quantify the complexity of the fault signal. Some of these methods are derived from information theory, including approximate entropy [10], sample entropy [11], and fuzzy entropy [12]. These methods have achieved some results in fault diagnosis, but each has its shortcomings [2,13]. Bandt and Pompe proposed the permutation entropy (PE), analyzing the complexity of time domain data by comparing neighboring values [14]. Compared with other nonlinear methods, PE does not rely on models. It has the advantages of simplicity, fast computation speed and good robustness [2,15]. However, the PE algorithm does not retain additional information in addition to the order structure when extracting the ordering pattern for each time series. Therefore, information that has a significant difference in amplitude will produce the same sort mode, which results in the calculated entropy value being inaccurate [15]. Fadlallah et al. proposed a modified PE method called weighted permutation entropy (WPE). WPE introduces amplitude information into the computation of PE by assigning the weight of the signal sorting mode [16]. The WPE method has been successfully applied to the dynamic characterization of electroencephalogram (EEG) signals [15], the complexity of stock time series [17], and so on. At the same time, a better differentiation effect is achieved than that of PE. In the field of mechanical fault diagnosis, a large number of literature calculates the PE value of the vibration signal as its status characterization [2,18,19]. On the other hand, the amplitude information of the bearing vibration signal is very critical for fault characterization, and cannot be ignored during fault feature extraction. However, there are few studies on the application of the WPE method to the analysis of vibration bearing signals.

In addition, recently, the research of Yan et al. [20] and Zhang et al. [2] shows that PE can effectively monitor and amplify the dynamic changes of vibration signals and characterize the working state of a bearing under different operating conditions. Combining the characteristics of the WPE and EEMD algorithms, this paper can effectively highlight the bearing fault characteristics under the multi-scale by calculating the WPE value of each IMF component decomposing from the original signal and forming the feature vector.

After the feature extraction, the classifier should be utilized to realize automatic fault diagnosis. Most machine learning algorithms, including pattern recognition and neural networks, require a large number of high-quality sample data [21]. In fact, bearing fault identification is controlled by the application environment. In reality, a large number of fault samples cannot be obtained. Therefore, it is crucial that the classifier can handle small samples and have good generalization ability. A support vector machine (SVM), proposed by Vapnik [22], is a machine learning method based on statistical learning theory and the structural risk minimization principle. Since the 1990s, it has been successfully applied to automatic machine fault diagnosis, significantly improving the accuracy of fault detection and recognition. Compared with artificial neural networks, SVM is very suitable for dealing with small sample problems, and has a good generalization ability. SVM provides a feasible tool to deal with nonlinear problems that is very flexible and practical for complex nonlinear dynamic systems. Besides, the combination of fuzzy control and a metaheuristic algorithm is widely used in the control of nonlinear dynamic systems. Bououden et al. proposed a method for designing an adaptive fuzzy model predictive control (AFMPC) based on the ant colony optimization (ACO) [23] and particle swarm optimization (PSO) algorithm [24], and verified the effectiveness in the nonlinear process. The Takagi–Sugeno (T–S) fuzzy dynamic model has been recognized as a powerful tool to describe the global behavior of nonlinear systems. Li et al. [25] deals with a real-time-weighted, observer-based, fault-detection (FD) scheme for T–S fuzzy systems. Based on the unknown inputs proportional–integral observer for T–S fuzzy models, Youssef et al. [26] proposed a time-varying actuator and sensor fault estimation. Model-based fault diagnosis methods can obtain high accuracy, but the establishment of a complex and effective system model is the first prerequisite. 

In addition, a large number of improved algorithms for SVM have been proposed. These algorithms focus on the parameter selection and training structure of SVM. Zhang et al. improved the bearing fault recognition rate by optimizing the parameters of SVM using the inter-cluster distance in the feature space [2]. In order to reduce the training and testing time of one-against-all SVM, Yang et al. proposed a single-space-mapped binary tree SVM; the other option is a multi-space-mapped binary tree SVM for multi-class classification [27]. Monroy et al. has developed a semi supervised algorithm, which combines a Gauss mixed model, independent component analysis, Bayesian information criterion and SVM, and effectively applies it to fault diagnosis in the Tennessee Eastman process [28]. The combination of supervised or unsupervised learning methods with SVM is a hot research focus in fault diagnosis. A large number of studies show that its performance has improved significantly more than the optimization of a single SVM [3,28,29].

Therefore, this study aggregated multiple SVM models into one combined classifier, abbreviated as an SVM ensemble classifier. When there are significant differences between the classifiers, the combined classifier can produce better results [30]. In addition, the essence of fault recognition is classification. In this study, a cloud similarity measurement (CSM) is introduced to quantify the similarity between vibration signals, which also provides the basis for the bearing fault identification [31]. At the decision-making stage, a hybrid voting (HV) strategy is proposed to improve the accuracy of recognition. The HV method is based on static weighted voting and the CSM algorithm. The final classification category is achieved through maximizing the output of the decision function.

This study presents the method based on WPE, EEMD and an SVM ensemble classifier for fault detection and the identification of rolling bearings. First, the WPE value of the vibration signal within a certain time window is calculated and the current working state of the bearing judged to detect whether the bearing is faulty. Then, if the bearing has a fault, the fault vibration signal is adaptively decomposed into a plurality of IMF components by the EEMD algorithm, and the WPE value of the first several IMF components is calculated as the feature vector of the fault. Next, the SVM ensemble classifier is trained using fault feature vectors. The ensemble classifier considers the classification results of a single SVM model and the similarity of the vibration signals with the decision function using the hybrid voting strategy. Finally, the fault detection and multi-fault recognition models will be verified using actual data. At the same time, the results of the fault recognition in this paper are compared with those published in other recent literature, as well as different decision rules and conventional ensemble classifiers.

The remaining part of this paper is organized as follows. In Section 2, the EEMD algorithm and its parameter selection is introduced and discussed. Section 3 introduces the PE and WPE. The structure, algorithm and CSM of the SVM ensemble classifier are detailed in Section 4. In Section 5 and Section 6, the steps and empirical research of the proposed fault diagnosis method are described in detail. In Section 7, a comparative study of current research and some literature is carried out, and limitations and future work are discussed. Finally, the conclusions are drawn in Section 8.

## 2. Ensemble Empirical Mode Decomposition

The EEMD algorithm is an improved version of the EMD algorithm. The essence of the EMD is to decompose the waveform or trend of different scales of any signal step-by-step, producing a series of data sequences with different characteristic scales. Each sequence is then regarded as an intrinsic mode function (IMF). All IMF components must satisfy the two following conditions: (1) the number of extreme points is equal to the number of zero points or differ at most by one in the whole data sequence; (2) the mean value of the envelope defined by the local maximum and local minimum is zero at any time. In other words, the upper and lower curves are locally symmetrical about the axis. However, the traditional EMD still suffers from the mode mixing problem that will make the physical meaning of individual IMFs unclear and affect the effect of the subsequent feature extraction.

Due to the introduction of white noise perturbation and ensemble averaging, the scale mixing problem is avoided for EMD, which allows the final decomposed IMF component to maintain physical uniqueness. EEMD is a more mature tool for non-linear and non-stationary signal processing [13]. Therefore, this study adopts EEMD to adaptively decompose bearing vibration signals to highlight the characteristics of the fault in each frequency band. The specific steps of the vibration signal decomposition process of rolling bearings based on the EEMD algorithm are as follows:

Step 1: Determine the number of ensemble *M* and the amplitude an of the added numerically-generated white noise.

Step 2: Superpose a numerically-generated white noise $n_{i}\left(t\right)$ with the given amplitude a on the original vibration signal *x*(*t*) to generate a new signal:

(1) $$x_{i}(t)=x(t)+n_{i}(t)$$

where $n_{i}\left(t\right)$ is a white noise sequence added to the *i*-th time, and $x_{i}\left(t\right)$ is a new signal obtained after the *i*-th superposition of white noise, while i=1,2,⋯,M.

Step 3: The new signal $x_{i}\left(t\right)$ is decomposed by EMD, and a set of IMFs and a residual component are obtained.

(2) $$x_{i}(t)=\sum_{s=1}^{S}C_{i,s}(t)+r_{i}(t)$$

where *S* is the total number of IMFs, and $r_{i}\left(t\right)$ is the residual component that is the mean trend of the signal. $\left[C_{i,\;1}(t),C_{i,\;2}(t),\cdots ,C_{i,\;S}(t)\right]$ represent the IMFs from high frequency to low frequency.

Step 4: According to the number of ensemble *M* sets in Step 1, repeat Step 2 and Step 3 *M* times to obtain an ensemble of IMFs.

(3) $$\left[\{C_{1,\;s}(t)\},\{C_{2,\;s}(t)\},\cdots ,\{C_{M,\;s}(t)\}\right]$$

Step 5: The ensemble means of the IMFs of the *M* groups is calculated as the final result:

(4) $$C_{s}(t)=\frac{1}{M}\sum_{i=1}^{M}C_{i,\;s}(t)$$

where $C_{s}\left(t\right)$ is the *s*-th IMF decomposed by EEMD, while i=1,2,⋯,M and s=1,2,⋯,S.

It is noteworthy that the number of ensemble *M* and the white noise amplitude a are two important parameters for EEMD, which should be selected carefully. In general, due to the EEMD algorithm being sensitive to auxiliary noise, the auxiliary white noise amplitude is usually small. In their original paper, Wu and Huang [9] suggested that the standard deviation of the amplitude for auxiliary white noise is 0.2 times that of the signal standard deviation. In addition, the amplitude of the auxiliary white noise should be reduced properly when the data is dominated by a high frequency signal. On the contrary, the noise amplitude may be increased when the data is dominated by a low frequency signal. On the other hand, the selection of the number of ensembles *M* determines the elimination level of the white noise added to the signal in the post-processing process. Indeed, the effect of the added auxiliary white noise on the decomposition results can be reduced by increasing the number of ensembles *M*. When the number of ensembles *M* reaches a certain value, the interference caused by the added white noise to the decomposition result can be reduced to a negligible level. Wu and Huang [9] suggested that an ensemble number of a few hundred will contribute to a better result. In the present study, the EEMD algorithm was used to decompose the vibration signals of the bearing. After many tests, it was found that proposed method with *M* = 100 and *a* = 0.2 led to a satisfying result. This conclusion is also consistent with some literature [2,13]. Therefore, this study set the parameters *M* = 100 and *a* = 0.2.

## 3. Weighted Permutation Entropy

### 3.1. Permutation Entropy and Weighted Permutation Entropy

Permutation entropy (PE) is a complexity measure for nonlinear time series, proposed by Bandt and Pompe in 2002 [14]. The main principle of PE is to consider the change of permutation pattern as an important feature of the time dynamics signal, and the entropy based on proximity comparison is used to describe the change in the permutation pattern.

Consider a time series {x(1),x(2),⋯,x(N)}, where *N* is the series length. The time series is reconstructed by phase space to obtain the matrix:

(5) $$X(j)=\left[\begin{array}{cccc} x(1) & x(1+\tau ) & \cdots & x(1+(m-1)\tau )\\ \vdots & \vdots & \ddots & \vdots \\ x(j) & x(j+\tau ) & \cdots & x(j+(m-1)\tau )\\ \vdots & \vdots & \ddots & \vdots \\ x(G) & x(G+\tau ) & \cdots & x(G+(m-1)\tau ) \end{array}\right]$$

where *m* ≥ 2 is the embedding dimension and τ is the time lag; *G* represents the number of refactoring vectors in the reconstructed phase space, while G=N−(m−1)τ. Rearrange the *j*-th reconstructed component in the matrix in ascending order:

(6) $$\left\{x(j+(i_{1}-1)\tau )\leq x(j+(i_{2}-1)\tau )\leq \cdots \leq x(j+(i_{m}-1)\tau )\right\}$$

If *X*(*j*) has the same element, it is sorted according to the size of the *i*. In other words, when $x(j+(i_{p}-1)\tau )=x(j+(i_{q}-1)\tau )$ and *p* ≤ *q*, the sorting method is $x(j+(i_{p}-1)\tau )\leq x(j+(i_{q}-1)\tau )$. Hence, any vector *X*(*j*) can get a sequence of symbols: $\pi_{i}=(i_{1},i_{2},\cdots ,i_{m})$. In the m dimensional space, each vector *X*(*j*) is mapped to a single motif out of *m*! possible order patterns $\pi_{i}$. For a permutation with number $\pi_{i}$, let $f(\pi_{i})$ denote the frequency of the *i*-th permutation in the time series. The probability that each symbol sequence appears is defined as:

(7) $$P(\pi_{i})=\frac{f(\pi_{i})}{\sum_{i=1}^{m!}f(\pi_{i})}$$

The permutation entropy of a time series is defined as:

(8) $$H_{p}(m)=-\sum_{i=1}^{m!}P(\pi_{i})\ln P(\pi_{i})$$

Obviously, $0\leq H_{p}(m)\leq \ln(m!)$, where the upper limit ln(m!) is $P(\pi_{i})=1/m!$. Normally, the *H_p_*(*m*) is normalized between [0, 1] through Formula (9).

(9) $$H_{p}=\frac{H_{p}(m)}{\ln(m!)}$$

**Figure 1:**
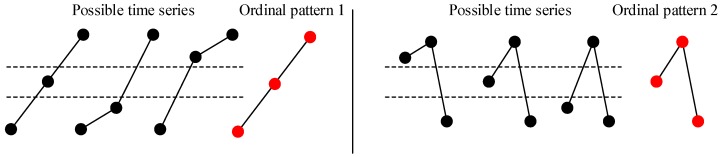
Two examples of possible time series corresponding to the same ordinal pattern.

PE provides a measure to characterize the complexity of nonlinear time series by the ordinal pattern. However, according to the definition, the PE neglects the amplitude differences between the same ordinal patterns and loses the information about the amplitude of the signal [32]. For the vibration signal, the amplitude contains a large amount of state information about the bearing, which should be one of the characteristics describing the running state. Figure 1 shows a case where different time series are mapped into the same ordinal pattern when the embedding dimension *m* = 3. The distances between the three points of different time series are not equal (the amplitude information of the signal is different). However, according to the PE algorithm, their ordinal patterns (1 and 2) are the same, resulting in the same PE value.

Fadlallah modified the process of obtaining the PE value, preserving useful amplitude information from the signal and proposing weighted permutation entropy (WPE) [16]. WPE is weighted for different adjacent vectors with the same ordinal pattern but different amplitudes. Hence, the frequency of the *i*-th permutation in the time series can be defined as:

(10) $$f_{\omega }(\pi_{i})=\sum_{s=1}^{S}f(\pi_{i}(s))\cdot \omega_{i}(s)$$

where s=1,2,⋯,S and *S* is the number of the possible time series in the same ordinal pattern. The weighted probability for each ordinal pattern is:

(11) $$P_{\omega }(\pi_{i})=\frac{f_{\omega }(\pi_{i})}{\sum_{i=1}^{m!}f_{\omega }(\pi_{i})}$$

Note that $\sum_{i}P_{\omega }(\pi_{i})=1$. The weight values $\omega_{i}(s)$ are obtained by:

(12) $$\omega_{i}(s)=\frac{1}{m}\sum_{k=1}^{m}{\left[x(j+(k-1)\tau )-\bar{X}(j)\right]}^{2}$$

where $\bar{X}(j)$ is the arithmetic mean:

(13) $$\bar{X}(j)=\frac{1}{m}\sum_{k=1}^{m}x(j+(k+1)\tau )$$

Finally, WPE is calculated as:

(14) $$H_{\omega }(m)=-\sum_{i=1}^{m!}P_{\omega }(\pi_{i})\ln P_{\omega }(\pi_{i})$$

Similarly, we standardize the values of WPE between 0 and 1 as $H_{\omega }$:

(15) $$0\leq H_{\omega }=\frac{H_{\omega }(m)}{\ln(m!)}\leq 1$$

### 3.2. Parameter Settings for WPE

**Figure 2:**
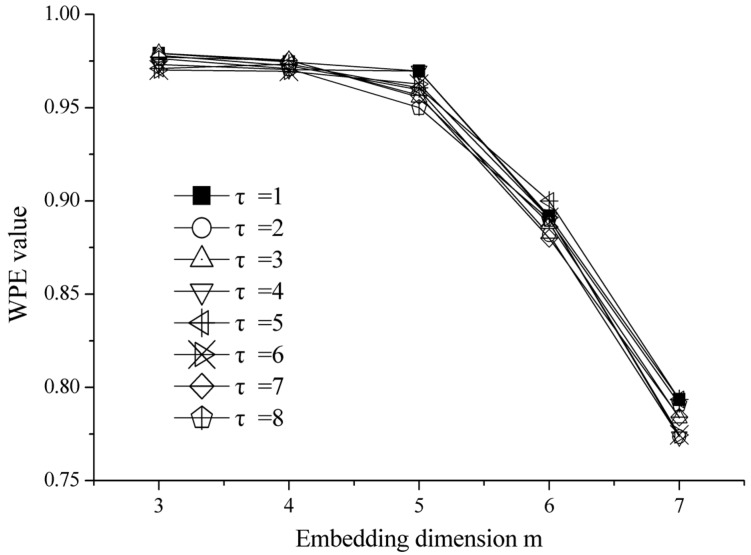
The effect of embedding dimension m and time lag τ on the Weighted Permutation Entropy (WPE) value.

Three parameters need to be set up before using WPE, including the embedding dimension m, the series length *N* and time lag τ. Bandt and Pompe [14] suggested that the value of *m* = 3, 4, …, 7. Cao et al. [33] recommended that *m* = 5, 6, or 7 may be the most suitable for the detection of dynamic changes in complex systems. Generally, this paper set *m* = 6 after testing. The time lag τ has little effect on the WPE value of the time series [2]. Taking one white noise time series with a length of 2400 points as an example, Figure 2 shows the curve of the WPE value with *m* = 3–7 change at different time lags τ = 1–8. Hence, according to the literature [2,13,14], the time lag τ is selected as 1. As for the series length *N*, it should be larger than the number of permutation symbols (*m*!) to have at least the same number of m-histories as possible symbols $\pi_{i}$, *i* = 1, 2, …, *m*!. Finally, this paper considers the sampling frequency of the bearing vibration signal and test to determine the series length *N* = 2400.

## 4. SVM Ensemble Classifier

### 4.1. Brief Introduction of SVM

SVM is proposed by Vapnik [22] and widely used in fault diagnosis. The principle of SVM is based on statistical learning theory. By converting the input space to a high-dimensional feature space, an optimal classification surface is found in the high-dimensional space. Under the premise of no error separation between the two types of samples, the classification interval is the largest and the real risk is the smallest.

Given a dataset with n examples (*x_i_*, *y_i_*), *i* = 1, 2, …, *n*, where $x_{i}\in R^{m}$ presents the *m* dimension input feature of the *i*-sample. $y_{i}\in \left\{+1,-1\right\}$ is the corresponding label for the input sample. The SVM method uses the kernel function $K\left(x_{i},x_{j}\right)$ to map the classification problem to a high dimensional feature space, and then constructs the optimal hyperplane *f*(*x*) in the transformed space.

$$f(x)=\mathrm{sgn}[\sum_{i=1}^{N_{SV}}a_{i}y_{i}^{SV}K(x_{i}^{SV},x)+b]$$

where, sgn is a sign function; *N^SV^* is the number of support vectors; $x_{i}^{SV}$ is the *i*-th support vector; $y_{i}^{SV}$ is the label of its corresponding category; $a_{i}\in R^{m}$ is the Lagrange multiplier; b∈R is the threshold; and *a_i_* can be solved by the following optimization problem.

$$\min\frac{1}{2}\sum_{i=1}^{N^{SV}}\sum_{j=1}^{N^{SV}}a_{i}a_{j}y_{i}^{SV}y_{j}^{SV}K(x_{i}^{SV},x)-\sum_{i=1}^{N^{SV}}a_{i}$$

$$\begin{array}{c} s.t.\;C\geq a_{i}\geq 0,i=1,2,\cdots ,l\\ \sum_{i=1}^{N^{SV}}a_{i}y_{i}^{SV}=0 \end{array}$$

In the form, *C* is the penalty factor. There are many kinds of kernel function $K\left(x_{i},x_{j}\right)$. However, in general, the radial basis function (RBF) is a reasonable first choice and thus is the most common kernel function of SVM [2]. In the study, the RBF kernel is used for kernel transformation and given as follows:

(16) $$K(x_{i}^{SV},x)=\exp(-\gamma {\left\|x-x_{i}^{SV}\right\|}^{2}),\;\gamma >0$$

where γ is the kernel parameter. In addition, there are two parameters (γ,C) that need to be optimized for SVM with the RBF kernel function. In the present study, *C* and γ are determined by the grey search method [34].

### 4.2. Multi-Class SVM and Ensemble Classifiers

A typical SVM is a binary classifier that can separate data samples into positive and negative categories. With real problems, however, we deal with more than two classes. For example, bearing failure conditions include inner race defects, outer race failure and roller element defects, etc. Accordingly, multi-class SVM is achieved by decomposing the multi classification problem into several numbers of binary classification problem. One method is to construct m binary classifiers, where m is the number of classes. Each binary classifier separates one of the classes from the other classes, which is called the one-against-all method (OAA). When using the OAA method to classify a new sample, we need to select a class with positive labels first and the other examples should have negative labels. Another way is to construct m(m−1)/2 classifiers; each classifier separates only two classes. For example, for the fault set $F=\left\{f_{1},f_{2},f_{3}\right\}$, 3×(3−1)/2=3 classifiers are needed to classify the binary set $\left\{f_{1},f_{2}\right\},$ $\left\{f_{1},f_{3}\right\}$ and $\left\{f_{2},f_{3}\right\},$ respectively. Next, majority voting rules are used to vote on the classification results of m(m−1)/2 SVM classifiers. The classification result with the highest number of votes will finally be selected. This method is more efficient than the OAA method, but has a major limit. Each binary classifier is trained by the data from only two types of sample. However, in the actual fault recognition process, the data that needs to be diagnosed may come from any class [21]. 

**Figure 3:**
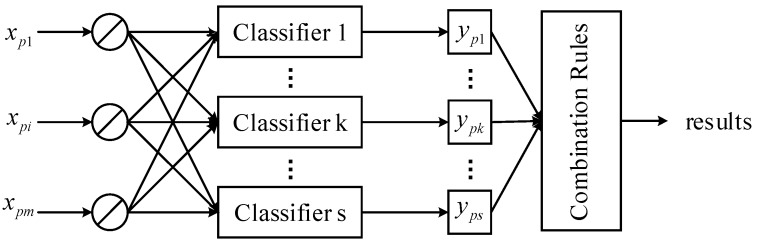
Structural schematic diagram of an ensemble classifier.

In order to solve this problem, the ensemble classifier is usually used to obtain a comprehensive result. Compared with a single classifier, different classifiers in ensemble classifiers can provide complementary information for fault classification, so that more accurate classification results can be obtained [21]. For the problem of multiple fault recognition, the target of the ensemble classifier is to achieve the best classification accuracy. The ensemble classifier combines the output of multiple classifiers according to some rules, and finally determines the category of a fault sample. A typical ensemble classifier is shown in Figure 3.

As shown in Figure 3, suppose the ensemble classifier consists of s classifiers. For the sample $x_{p}\in R^{m}$, each classifier k has an actual output $y_{pk}\in \left\{-1,1\right\}$. Then, to achieve the best classification accuracy rate, the outputs of multiple classifiers s are combined according to a certain rule, and the final results are determined.

### 4.3. Multi-Fault Classification Based on an SVM Ensemble Classifier

An SVM ensemble classifier is a new combination strategy based on SVM. The multiple fault models are generated using SVMs to learn all fault data with the related data set. Each SVM model is used to classify the newly obtained bearing vibration signals, and their respective results are combined to obtain higher fault identification performance. 

The SVM ensemble classifier algorithm is mainly divided into two phases: the training of single classifiers and the combination of multiple classifiers according to the relevant rules. For fault *f_i_*, the training samples for the SVM model are *x*_1_, *x*_2_, …, *x_i_*, …, *x_n_*; *y*. Among them, *x_i_* is the bearing state characteristic value; *y* is the category label (including the bearing normal state and the fault state). The process of building the single fault classification model *f*(*x*) is as follows [21]:

(1) Standardization of the training sample set (*x*_1_, *x*_2_, …, *x_i_*, …, *x_n_*, *y*).
(2) Use RBF as the kernel function of the SVM and optimize the SVM parameters with cross-validation method (CV) [34].
(3) Calculate the Lagrange coefficient $a_{i}$.
(4) Obtain the support vector sv().
(5) Calculate the threshold *b*.
(6) Establish an optimal classification hyperplane *f*(*x*) for training samples.

**Figure 4:**
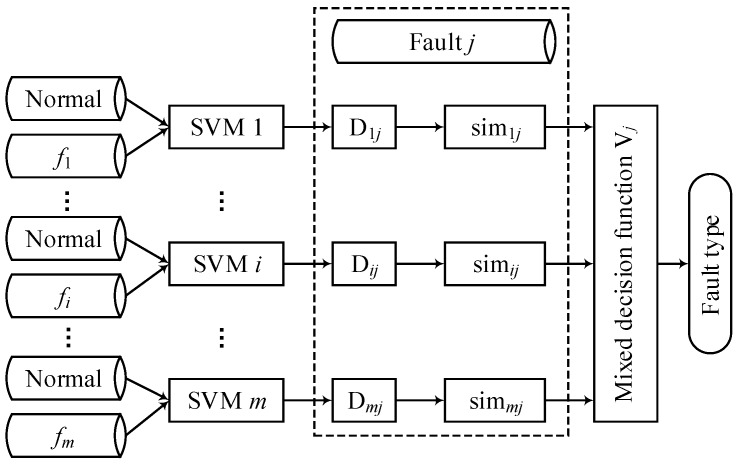
SVM ensemble classifier based on a mixed decision.

In ensemble classifiers, a single fault classification model is a base classifier. The literature shows that the base classifier needs to satisfy the diversity and accuracy in order to achieve a better integration effect [35]. The most common way to introduce diversity into classifiers is to deal with the distribution or feature space of training samples. The single fault model in this study was trained using normal operating and different fault bearing vibration data (Figure 4), using differently-distributed training samples to produce different base classifiers. At the same time, the accuracy of the base classifier is guaranteed by calculating the classification error rate.

The multiple fault classification model *g*(*x*) is derived from the single fault model *f*(*x*) according to the relevant rules. It is obvious that the contribution of the different single fault models to the final fault identification results is different. Hence, how to set the weight of each single SVM classifier is a key issue for the SVM ensemble classifier. A common method is to assign weights based on the classification accuracy of each classifier [36]. Assuming that the classification accuracy of the single fault model *f*(*x*) is *q_i_*, then the weight $\omega_{i}$ of *f*(*x*) in the multi fault model *g*(*x*) is as follows.

(17) $$\omega_{i}=\log\frac{q_{i}}{1-q_{i}}$$

Define the decision function *D_ij_*, which returns the classification result of the model *f*(*i*) for the category with the *j*-th data.

(18) $$\begin{array}{l} D_{ij}={\left(d_{1},d_{2},\cdots ,d_{i},\cdots ,d_{m}\right)}^{T}\\ d_{i}=\left\{ \begin{array}{ll} 1, & if\;the\;result\;of\;f(i)\;is\;f_{i}\\ 0, & else \end{array}\right. \end{array}$$

The decision function *D_ij_* is only the classification result from the single fault model *f*(*i*). Samples with similar fault states have similar vibration signals [37]. Therefore, the similarity between the *j*-th data and historical failure data should be considered in the multi-fault model. In this study, the similarity measurement between two vibration signals is quantified by cloud similarity measurement (CSM). CSM consists of a backward cloud generator algorithm and includes the angle cosine of the cloud eigenvector [31]. For the input sample set $A_{j}=(a_{1},a_{2},\cdots ,a_{N})$ and sample set $B_{k}=(b_{1},b_{2},\cdots ,b_{M})$, where N and M are the number of *A_j_* and *B_k_*, the CSM steps are as follows [31].

(1) Calculate the universal mean $\bar{A}=\left(1/n\right)\overset{n}{\underset{j=1}{\sum }}A_{j}$, the first order of sample absolute center distance $\left(1/n\right)\overset{n}{\underset{j=1}{\sum }}\left|A_{j}-\bar{A}\right|$ and sample variance $S^{2}=\left[1/\left(n-1\right)\right]\overset{n}{\underset{j=1}{\sum }}{\left|A_{j}-\bar{A}\right|}^{2}$ for data set *A_j_*.

(2) Calculate the universal mean $E_{A}$ of the cloud model.

(19) $$E_{A}=\bar{A}$$

(3) Calculate the feature entropy $E_{n}$ of the data *A_j_*.

(20) $$E_{n}=\sqrt{\frac{\pi }{2}}\cdot \frac{1}{n}\sum_{j=1}^{n}\left|A_{j}-E_{A}\right|$$

(4) Calculate the feature hyperentropy $H_{e}$.

(21) $$H_{e}=\sqrt{S^{2}-E_{n}{}^{2}}$$

(5) The digital features $E_{A}$, $E_{n}$ and $H_{e}$ are used to describe the overall characteristics of the vibration signals. The cloud vector of *A_j_* is $\vec{\upsilon_{j}}=\left(E_{Aj},\;E_{nj},H_{ej}\right)$; Similarly, the cloud vector of the reference sample *B_k_* is $\vec{\upsilon_{k}}=\left(E_{Ak},\;E_{nk},H_{ek}\right)$. The similarity of any two samples *A_j_* and *B_k_* may be described quantitatively by the included angle cosine between $\vec{\upsilon_{j}}$ and $\vec{\upsilon_{k}}$.

(22) $$sim_{jk}=\cos({\vec{\upsilon }}_{j},{\vec{\upsilon }}_{k})=\frac{{\vec{\upsilon }}_{j}\times {\vec{\upsilon }}_{k}}{\left\|{\vec{\upsilon }}_{j}\right\|\left\|{\vec{\upsilon }}_{k}\right\|}$$

Obviously, *sim_jj_* = 1 and *sim_jk_* = *sim_kj_*. Therefore, this paper defines the mixed decision function *V_j_* of the multi fault classification model as follows.

(23) $$V_{j}=\max(\sum_{i=1}^{m}D_{ij}\cdot \omega_{i}+sim_{ij})$$

The algorithm of the bearing multi-fault classification model *g*(*x*) is described below.

(1) Generate a single model *f*(*i*) for each fault *f_i_* using related data.
(2) Use Formula (17) to determine the weight $\omega_{i}$ of each model *f*(*i*).
(3) Calculate the decision functions *D_ij_* of the *j*-th fault data using model *f*(*i*).
(4) The final classification results of the *j*-th fault data are determined by SVM ensemble classifier with maximizing *V_j_*.

The classification mechanism of the SVM ensemble classifier is shown in Figure 4.

The single fault model in Figure 4 is trained by the vibration data of the normal running conditions and different fault conditions of the bearing. The unknown fault type *j* is the training data set, and finally the outputs of the fault type *j*.

## 5. Proposed Fault Diagnosis Method

Based on the EEMD, WPE and SVM ensemble classifier, a novel rolling bearing fault diagnosis approach is presented in present study, which mainly includes the following steps:

(1) Collect the running time vibration signals of the rolling element bearing.
(2) Decompose the vibration signal into the non-overlapping windows of the series length *N*.
(3) Use Formulae (11) and (14) to calculate the WPE values for the vibration signal.
(4) Fault detection is realized according to the WPE value of the vibration signal, which determines whether the bearing is faulty. If there is no fault, output the fault diagnosis result that the bearing operation is normal and end the diagnosis process. If there is a fault, go to the next step.
(5) The collected vibration signal is decomposed into a series of IMFs using the EEMD method, and the WPE values of the first several IMFs are calculated as feature vectors using Equations (11) and (14).
(6) Input the feature vectors to the trained SVM ensemble classifier to get the fault classification result and output the fault type.

**Figure 5:**
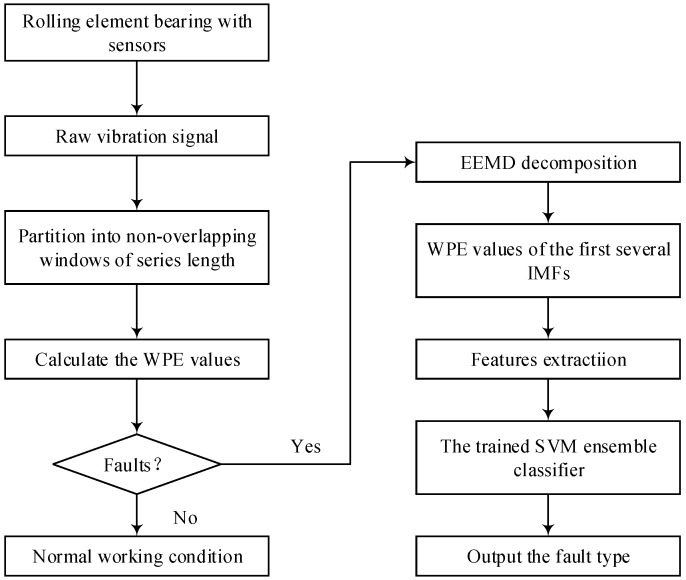
Flowchart of the proposed method.

The flow chart of multiple fault diagnosis for the bearings is shown in Figure 5.

## 6. Experimental Validation and Results

### 6.1. Experimental Device and Data Acquisition

**Figure 6:**
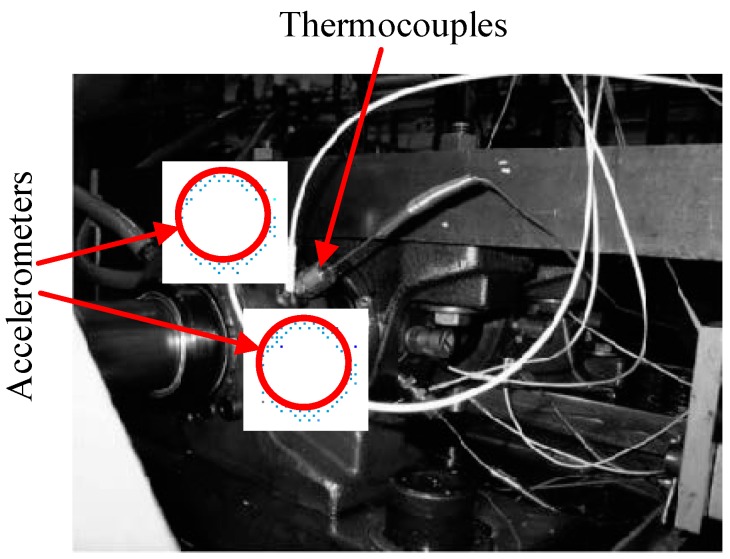
Schematic diagram of the sensor installation location.

**Figure 7:**
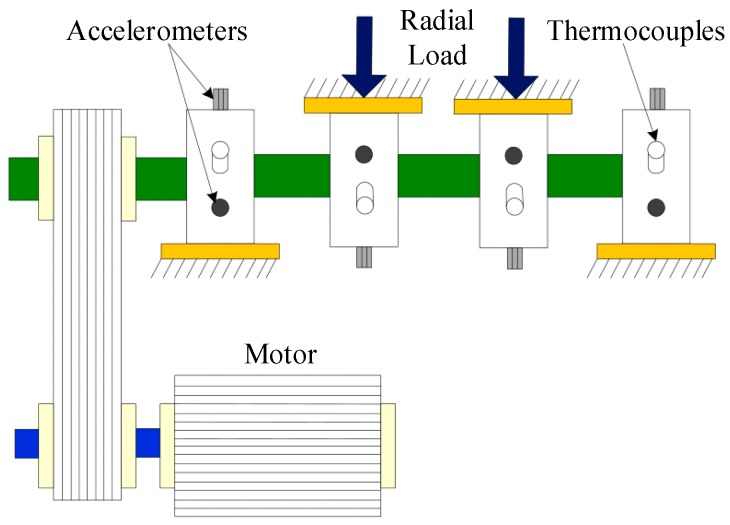
Structural diagram of the bearing test bench.

In order to verify the method of rolling bearing fault diagnosis proposed in present study, experimental data were applied to test its performance. The data set was kindly provided by the University of Cincinnati [38]. The installation position of the sensor and structure of the bearing test bench are shown in Figure 6 (the acceleration sensors are in the circle) and Figure 7.

As shown in Figure 6, four double-row cylindrical roller test bearings were mounted on the drive shaft. Each bearing had 16 rollers with a pitch diameter of 2.815 inches, a ball diameter of 0.331 inches and a contact angle of 15.17 degrees. The rotation speed of the shaft was kept constant at 2000 revolutions per minute (RPM). Furthermore, a radial load of 6000 lbs was imposed on the shaft and bearing by the spring mechanism. A high sensitivity Integrated Circuit Piezoelectric (ICP) acceleration sensor was installed on each bearing (see Figure 7). The magnetic plug is installed in the oil return pipeline of the lubricating system. When the adsorbed metal debris reaches a certain value, the test will automatically stop, the specific fault of the bearing will then be stopped and a new bearing installed for the next group of tests. The sampling rate was set to 20 kHz, and each 20,480 data points were recorded in one file. Data were collected every 5 or 10 min, and the data files were written when the bearing was rotated.

**Figure 8:**
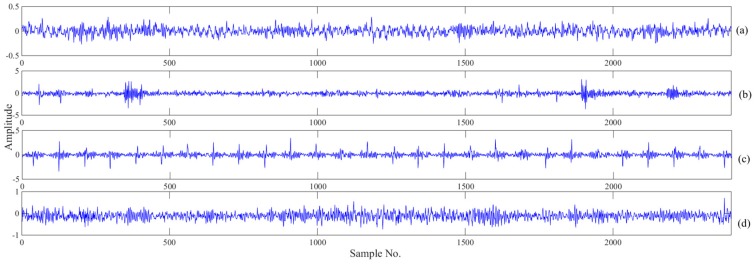
Vibration signals of each bearing condition. (**a**) normal, (**b**) inner race fault (IRF), (**c**) outer race fault (ORF), (**d**) roller defect (RD).

Four kinds of data including normal data, inner race defect data, outer race defect data and roller defect data were selected in the present study. Each data file contains 20,480 data points. Considering the series length *N* for the WPE value, a single data file cannot be directly used as the calculation input. Hence, the data will be segmented into segments and form the sample sets. The sampling frequency of the vibration signal was 20 kHz, and the rotating speed of the bearing was 2000 RPM. Thus, a rotation period can be calculated to contain 600 data points. The size of the segmentation was set to be three times that of the rotation period, which was 2400 data points. In other words, each data file can be divided into at least eight sample sets. For each bearing running state, 600 sample sets were selected in this study. Figure 8 shows the vibration signals of normal bearings and faults in three rotation periods. 

As shown in Figure 8, the four running states of the bearing show a similar trend, and it is difficult to identify and classify them by intuition. Therefore, it is necessary to classify them with appropriate mathematical model methods. The details of the dataset are shown in Table 1.

### 6.2. Fault Detection

**Figure 9:**
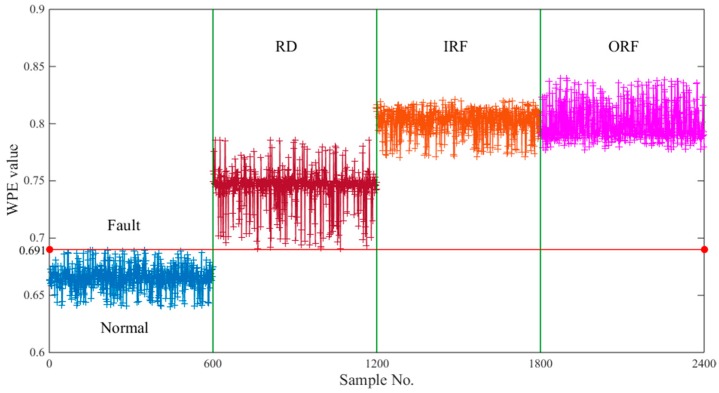
The WPE value of all the samples: the first 600 samples are in normal working condition and their WPE values are lower than 0.691. The rest of the 1800 samples are in defective working condition with roller defect, inner race fault and outer race fault, respectively.

Bearing fault detection is a prerequisite for bearing fault classification. The WPE values of the 2400 samples in Table 1 were calculated in turn and shown in Figure 9. Obviously, we can observe that the normal sample and the faulty sample are clearly separated. When the WPE value is greater than 0.691, a fault occurs in the bearing operation. On the contrary, the bearing state is in normal working condition. These facts show that the WPE value of the bearing vibration signal can be used as a fault detection standard to achieve bearing fault detection. However, there is an intersection of WPE values for different faults. The WPE value cannot be used as a standard for fault classification. Faults need further identification and classification.

The samples with fault have a larger WPE value, which indicates that they are more complex than the normal sample in the vibration signal. When the bearings are running normally, the vibration mainly comes from the interaction and coupling between the mechanical parts and the environmental noise, and the vibration signal has a certain regularity. As a result, the WPE values are smaller than those in comparison. When the bearing is faulty during operation, the fault characterized by the impulses will introduce some impulsive components. The high frequency vibration mixed with the vibration signal of the bearing makes the vibration signal more complex with wide band frequency components. 

Fault detection is the first step of fault diagnosis. For a complex system, it is necessary to detect the fault sensitively, and then classify and identify the faults. If the fault is not detected, the result of the system is in normal working condition. 

### 6.3. Fault Identification

**Figure 10:**
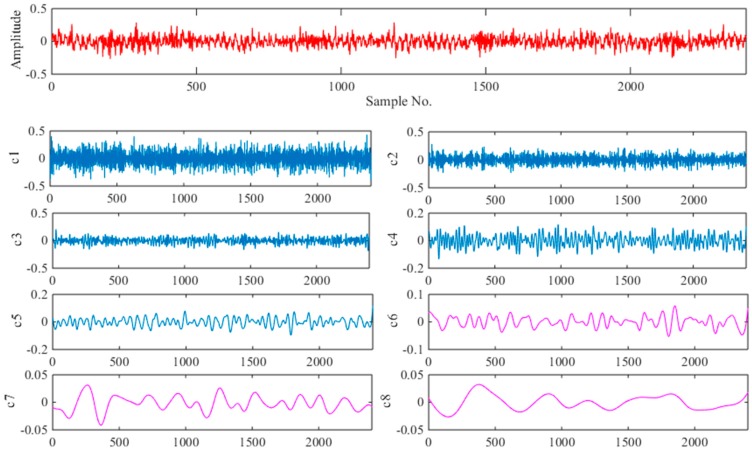
Decomposed signal in normal working condition using EEMD.

**Figure 11:**
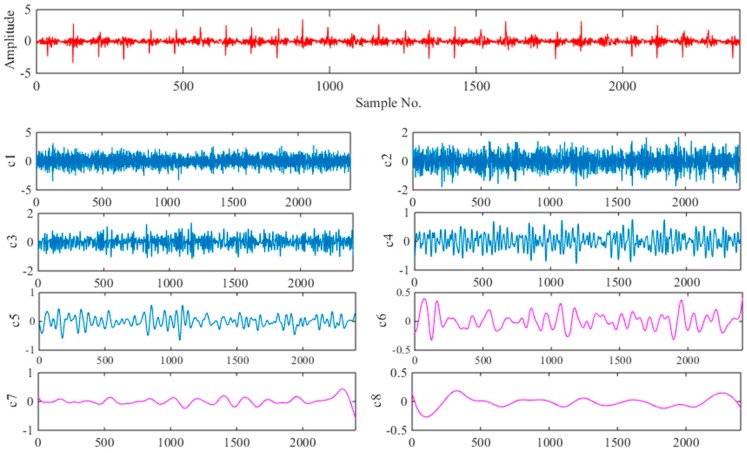
Decomposed signal with outer race fault using EEMD.

When faults are detected, the SVM ensemble classifier is used for fault classification. First, each sample is decomposed by EEMD algorithm. According to the discussion in Section 2, the two parameters (*M*, *a*) of the EEMD were set as *M* = 100, *a* = 0.2. Figure 10 and Figure 11 show the time-domain graph with normal working conditions and outer race fault, respectively, including their EEMD decomposition results for the signal containing the first eight IMFs. Intuitively, the IMF components decomposed from the vibration signals collected in different states have obvious differences. Compared with the original signal, IMFs can display more feature information.

**Figure 12:**
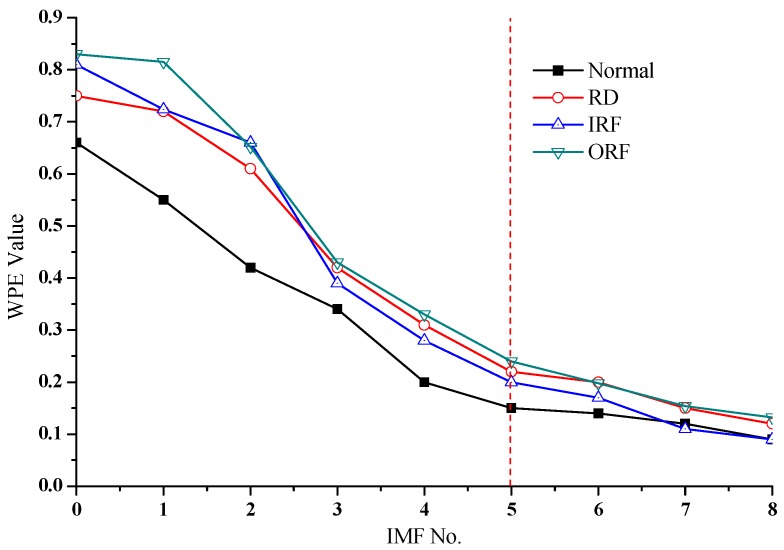
WPE values of IMFs under different fault modes.

EEMD decomposition was performed on the data in the four state types. Next, we selected the WPE parameter *m* = 6, τ=1, and calculated the WPE value of the first eight IMFs obtained by decomposition in each state. Figure 12 shows the WPE values of the original signal and the corresponding EEMD decomposed signal. Among them, the IMF number 0 represents the original signal, and 1–8 represent the decomposed IMFs.

As can be seen from Figure 12, the WPE values of different state data and their decomposed IMFs are different. Compared with the normal operating state of the bearing, the vibration signal of the fault state and their first several IMFs have larger WPE values. The vibration signals will appear due to some high-frequency pulses caused by the fault; as a result, the complexity of the first several IMFs decomposed from the signal will increase. Besides, Figure 12 can also observe the distribution of different fault data with different WPE values decomposed by EEMD. Therefore, the WPE value of IMFs can describe the working state of the bearing.

On the other hand, in Figure 12, we found that the first five IMFs almost contain the most important information of the signal. The WPE values of these IMFs are quite different, and their contribution to fault classification is also the largest. In contrast, the WPE values of the later IMFs are almost the same, which contributes litter to the fault classification. Hence, the present study calculates the WPE values of the first five IMFs for each sample by EEMD decomposition as the feature vectors of fault information. 

In this study, the purpose of multiple fault recognition models is to distinguish RD, IRF and ORF. Therefore, first we needed to build three single fault models. As a result, a single fault classification model was established by selecting RD data, IRF data and ORF data separately from normal state data. Two-thirds of the data set was the training set, and the rest was the test set. Next, the value of qi and the weight $\omega_{i}$ for the single fault model were determined. In addition, an SVM ensemble classifier was used to identify multiple faults. The similarity measurement between two vibration signals was quantified by CSM, and the reference sample was selected randomly from the fault data set. Each experiment was repeated 10 times. The classification accuracy (CA) and the variance of the CA were finally reported.

$$\mathrm{CA}=\frac{number\;of\;correctly\;classified\;samples}{total\;number\;of\;samples\;in\;dataset}\times 100\%$$

The classification results of each fault and total fault in the testing processes are presented in Table 2 and Table 3. 

The accuracies and variances of the three classifications are measured separately. Roller defect faults are the easiest to identify, because no matter how the model parameters change, its classification result is always correct. There are defects in the classification of IRF data and ORF data, but most of them can be separated by the SVM ensemble classifier. Furthermore, the confusion matrix is a useful tool to study how classifiers identify different tuples, which contains information on the actual classification and the prediction classification performed by the classification system. From Table 2 and Table 3, it can be seen that the SVM ensemble classifier can effectively identify the defect sample, especially for the RD vibration signal. The recognition result of the SVM ensemble classifier is ideal because its overall classification accuracy rate is close to 97.78%.

**Figure 13:**
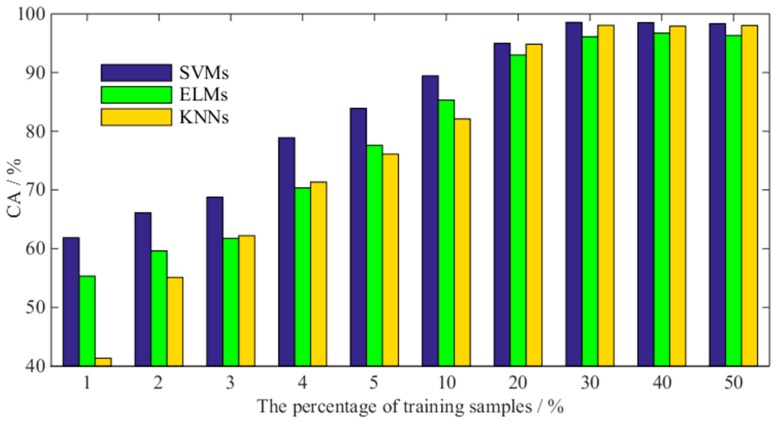
Classification accuracy under different percentages of training data sets: from left to right, the SVMs, ELMs, and KNNs classifiers are in turn.

The SVM ensemble classifier uses the optimal strategy of the decision function to improve the accuracy of fault identification. In fact, there are a large number of classification methods that can be used as classifiers, such as neural networks, decision trees, and K-nearest neighbor classification methods. Why is only the SVM classifier used for the SVM ensemble classifier? As mentioned in the introduction, SVM has an advantage when dealing with small sample data. Hence, the present study compares the average classification accuracy of SVM, extreme learning machine neural network (ELM) and the K-nearest neighbor method (KNN) under different proportions of training samples. The training data and test data were randomly selected in each experiment, and the experiment was repeated 10 times. Under different types of classifiers, the average CA of the three types of fault is shown in Figure 13.

As shown in Figure 13, with the decrease of the percentage of the training set data, the average CA values of the multi-classification models constructed by the above three classifiers decreased. However, the CA of the SVM ensemble classifier is always higher than that of other classifiers. Especially when the proportion of training samples is less than 20%, the SVM ensemble classifier has obvious advantages. In practice, the proportion of bearing fault data is very small. Therefore, the SVM model proposed in this paper has better practicality.

## 7. Discussion

### 7.1. Comparison of Different Decision Rules

Among the classifier fusion methods, the majority voting (MV) method is the most commonly used. The basic idea is to count the voting results of each classifier, and the category with the largest number of votes is the final category of the samples to be tested. In addition, another more in-depth method is the weighted voting (WV) rule, which is divided into static weighted voting (SWV) and dynamic weighted voting (DWV) according to the determination stage of the weight value. The weights of the SWV are determined in the training stage, while the weights of the DWV are determined in the testing stage and vary with the changes of input samples. Based on the SWV, CSM was introduced to quantify the similarity between the test samples and historical fault data, and a hybrid voting (HV) strategy was proposed in present study. Table 4 shows the difference in accuracy and variance between the above four decision rules. The training and testing samples were selected randomly from the data set. Each experiment was repeated 10 times. MV and WV integrate the results of classifiers without considering the similarity, and the base classifier is determined by one-against-one (OAO) method [2]. If an indistinguishable event occurs during the voting process, the experiment is repeated until 10 experiments have been completed. In order to demonstrate the effectiveness of the HV, the weights of SWV are calculated according to Formula (17), and the process of DWV is referred to in the literature [39].

As can be seen from the table, the MV performance was the worst, and the SWV was worse than the DWV. In contrast, HV had the highest classification accuracy with the lowest variance. The HV method consists of two parts: the base SVM classifier and the similarity calculation, and the testing samples are determined by the maximization of the decision function. In contrast to the conventional OAO method, the SVM classifier in HV only classifies normal samples and different fault samples, but does not include the classification between faults and faults. Therefore, for the MV and WV methods, there will be a case where the number of votes is the same and cannot be classified. On the other hand, the number of base classifiers is (n−1) times that of HV, and n is the total number of faults. In information theory, the HV method inputs more decision-making information, considering both the results of base classifier and the similarity with historical fault samples, rather than being a simple classification process.

### 7.2. Comparison with Conventional Ensemble Classifiers

**Figure 14:**
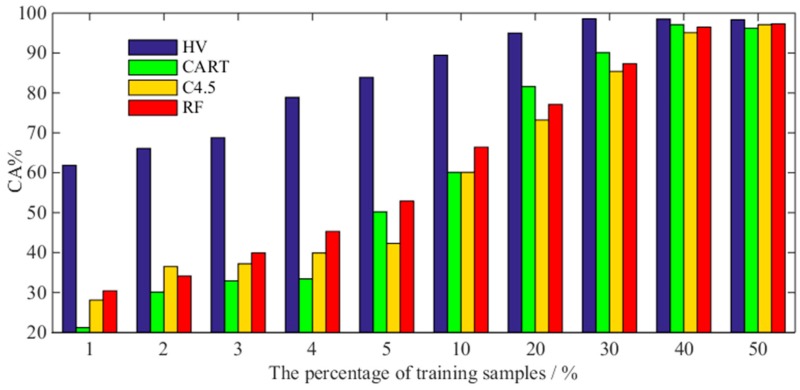
Classification accuracy of different ensemble classifiers under different percentages of training samples: from left to right, HV, CART, C4.5 and RF.

If the base classifier is compared to a decision-maker, the ensemble learning approach is equivalent to multiple decision-makers making a common decision. The ensemble learning algorithm is a popular algorithm in data mining technology. Since its birth, many conventional and classical algorithms have been used, such as CART, C4.5 [40], random forest (RF) [41], and so on. In Section 6.3, we discussed the classification accuracy of training samples with different proportions under different base classifiers. Similarly, Figure 14 compares the performance differences between the three conventional ensemble classifiers (CART, C4.5 and RF) and the HV method. In addition, the CART, C4.5 and RF methods were implemented using the algorithm provided by the Weka machine learning toolbox, and their parameters were all set by default in the toolbox. For example, the minimum number of samples for each leaf node of the C4.5 and CART algorithms was 2; the pruning ratio of C4.5 was set to 25%, and the number of trees in the random forest was 300. 

When the number of training samples is sufficient (the proportion is greater than 30%), the CA of different ensemble classifiers is almost the same. However, when the proportion of training samples decreases rapidly, the CA of the above three types of traditional ensemble classifiers also decreases sharply. The CART, C4.5 and RF algorithms still cannot escape the fact that a large number of training samples are needed.

### 7.3. Comparison with Previous Works

In order to illustrate the potential application of the proposed method in bearing fault identification, Table 5 presents a comparative study of present work and recently published literature using different methods. The comparative study items include feature extraction, feature selection methods, classifier type, number of fault type, construction strategy of training data set and final average classification accuracy (CA). 

The present study selected some of the literature that randomly selected data sets to construct training and test datasets for comparative study. It can be seen that the fault diagnosis method proposed in this paper has a higher accuracy of fault identification, which can be well applied to the fault diagnosis of bearings and meet the actual needs.

### 7.4. Limitations and Future Work

The work presented in this paper describes a rolling bearing fault detection and fault recognition method, and used real rolling element bearing fault data provided by the University of Cincinnati to verify its effectiveness. The results show that the proposed fault diagnosis method is in the same grade as recently published articles in terms of classification accuracy. In addition, compared to the traditional ensemble classifiers, the proposed method can also maintain a high classification recognition rate when there are few training samples. However, the proposed method still has limitations in several aspects. On the one hand, high-quality data sources are necessary. The type of data determines the selection and performance of the SVM kernel functions. On the other hand, the base classifier in the proposed method only uses normal data sets under the same situation and different fault data sets for training. In reality, external factors such as working conditions and materials can influence the vibrations of normal operation. In other words, the vibration signals of normal bearings are varied, and only one case is considered in the study. More importantly, the present study only discusses the fault conditions at a constant angular velocity of the bearing, and more complex variable angular velocity conditions and even the fault types of the different damage degrees still need to be further verified. Theoretically, the proposed method can arbitrarily increase the number of base classifiers to face different faults under more conditions. The SVM-based classifier itself can distinguish between normal samples and fault samples. It is possible to establish fault classifiers with different degrees of damage at a variable angular velocity, and then combine the CSM to quantify the similarity to achieve an extension of more complex situations. 

In addition, the experimental device at the University of Cincinnati used a belt transmission from the motor to the shaft. The belt can act as a mechanical filter of some acceleration frequencies and also generate frequencies not related to the bearing faults. Due to its own characteristics, the belt is a low-frequency vibration. In the transmission process, the belt can filter the vibration signal from the motor to the shaft. In addition, it also affects the vibration of the shaft. A belt drive system is a complex power device. For bearing fault diagnosis, the external noise or the disturbance of the power system is unfavorable. Meanwhile, the influence of the belt transmission on the fault signal is different under different motor speeds, which will further affect the experimental results. The instability of the belt will directly affect the results of the bearing fault diagnosis. A single shaft experimental set up may avoid this limitation [44], and fault feature extraction algorithm, which effectively processes fault features and external noise, will be the focus of future research. The early failure of a bearing is usually characterized by a low frequency vibration, and it is also necessary to verify the influence of belt transmission throughout the whole life degradation experiment. 

More attention will be paid to the limitations of the current research for future work, with a focus on the quantification of the similarity between the signal to be classified and the historical fault signal in the proposed model, as well as the method of selecting historical fault signals. Furthermore, a data-driven approach combining the knowledge-based and the physics-model-based method is the key to further research.

## 8. Conclusions

A method of rolling bearing fault diagnosis based on a combination of EEMD, WPE and SVM ensemble classifier was proposed in present study. Among them, a hybrid voting strategy was adopted in the SVM ensemble classifier to improve the accuracy of fault recognition. The main contribution of the hybrid voting strategy is that it not only combines the voting results of the base classifier, but also takes full account of the similarity between the samples to be classified and the historical fault data. A decision function is added to the ensemble classifier to comprehensively consider various aspects of classification information. 

The WPE algorithm has significant advantages in quantizing the complexity of the signal. In the study, the WPE value was used, on the one hand, to detect the fault of the bearing. On the other hand, when a fault occurred, the WPE value of the IMF component decomposed by the EEMD was calculated and constituted the fault feature vectors. The SVM ensemble classifier consists of a number of binary SVM classifiers and a decision function. The decision function takes into account the classification results of the binary SVM and the similarity between the vibration signals, and the result of the fault classification is synthesized. Finally, the fault diagnosis method was verified by the bearing experimental data from Cincinnati. The experimental results showed that the fault diagnosis method can accurately monitor bearing faults and identify the RD, IRF and ORF. Compared with the recent data-driven fault diagnosis method, the method of this paper has a higher accuracy of fault identification. In addition, the SVM ensemble classifier is very suitable for the classification of small sample data. When the proportion of training data sets decreases, it still maintains a good fault recognition effect. Moreover, the present work summarizes some of the limitations of the current research and illustrates concerns for future work, which will further verify the proposed method as well as improving the accuracy and robustness of fault recognition.

## Figures and Tables

**Table 1:** Description of the bearing data set.

Bearing Condition	Number of Training Data	Number of Testing Data
Normal	400	200
IRF	400	200
ORF	400	200
RD	400	200

**Table 2:** Confusion matrix of the multiclass SVM ensemble classifier resulting from the testing dataset.

Actual Classes	Predicted Classes		
	RD	IRF	ORF
RD	600	0	0
IRF	3	583	14
ORF	2	21	577

**Table 3:** The testing accuracy for different bearing conditions using the SVM ensemble classifier.

Fault Type	Average CA	Variance
RD	100%	0
IRF	97.17%	1.02
ORF	96.16%	0.37
Total	97.78%	0.12

**Table 4:** Comparison of the accuracy and variance of different decision rules.

Decision Rule	Average CA	Variance
MV	72.94%	1.13
SWV	78.56%	2.21
DWV	85.22%	2.45
HV	97.78%	0.12

**Table 5:** Comparisons between the present study and some published work.

Reference	Characteristic Features	Classifier	Number of Classified States	Construction Strategy of Training Data Set	CA (%)
Zhang et al. [42]	Divide time series data into segmentations	Deep Neural Networks (DNN)	4	Random selection	94.9
Yao et al. [43]	Modified local linear embedding	K-Nearest Neighbor (KNN)	4	Random selection	100
Saidi et al. [34]	Higher order statistics (HOS) of vibration signals + PCA	SVM-OAA	4	Random selection	96.98
Tiwari et al. [5]	Multi-scale permutation entropy (MPE)	Adaptive neuro fuzzy classifier	4	Random selection +10-fold cross validation	92.5
Zhang et al. [13]	Singular value decomposition	Multi class SVM optimized by inter cluster distance	3	Random selection	98.54
Present work	Weighted permutation entropy of IMFs decomposed by EEMD	SVM ensemble classifier + Decision function	3	Random selection	97.78
